# ZnO Interactions with Biomatrices: Effect of Particle Size on ZnO-Protein Corona

**DOI:** 10.3390/nano7110377

**Published:** 2017-11-06

**Authors:** Jin Yu, Hyeon-Jin Kim, Mi-Ran Go, Song-Hwa Bae, Soo-Jin Choi

**Affiliations:** Department of Applied Food System, Major of Food Science & Technology, Seoul Women’s University, Seoul 01797, Korea; ky5031@swu.ac.kr (J.Y.); kimhj043@naver.com (H.-J.K.); miran8190@naver.com (M.-R.G.); songhwa29@naver.com (S.-H.B.)

**Keywords:** zinc oxide, interaction, gastrointestinal fluid, plasma, particle size

## Abstract

Zinc oxide (ZnO) nanoparticles (NPs) have been widely used for food fortification, because zinc is essential for many enzyme and hormone activities and cellular functions, but public concern about their potential toxicity is increasing. Interactions between ZnO and biomatrices might affect the oral absorption, distribution, and toxicity of ZnO, which may be influenced by particle size. In this study, ZnO interactions with biomatrices were investigated by examining the physicochemical properties, solubility, protein fluorescence quenching, particle–protein corona, and intestinal transport with respect to the particle size (bulk vs. nano) in simulated gastrointestinal (GI) and plasma fluids and in rat-extracted fluids. The results demonstrate that the hydrodynamic radii and zeta potentials of bulk ZnO and nano ZnO in biofluids changed in different ways, and that nano ZnO induced higher protein fluorescence quenching than bulk ZnO. However, ZnO solubility and its intestinal transport mechanism were unaffected by particle size. Proteomic analysis revealed that albumin, fibrinogen, and fibronectin play roles in particle–plasma protein corona, regardless of particle size. Furthermore, nano ZnO was found to interact more strongly with plasma proteins. These observations show that bulk ZnO and nano ZnO interact with biomatrices in different ways and highlight the need for further study of their long-term toxicity.

## 1. Introduction

Zinc oxide (ZnO) is widely utilized in industry because of its ultraviolet (UV) protective, nutritional, and anti-microbial properties [1,2,3]. ZnO nanoparticles (NPs) are currently used in cosmetics, sunscreen products, the agricultural industry, food additives, and packaging [4,5]. In particular, ZnO NPs have been used as food fortifications and agricultural fertilizers, because zinc plays an important role in the metabolism and protein synthesis, and in the regulation of gene expression and enzyme and hormone activities [6,7,8]. However, NPs have a large surface area to volume ratio, which results in high reactivity and behaviors unlike those of micro-sized materials in biological systems. Hence, NPs might induce unexpected biological responses and biokinetic behaviors, and this raises public concerns about their potential toxicity.

Many studies have been performed on the toxicity of ZnO NPs in cell lines and animal models [9,10,11,12], and some conflicting results have been reported. The use of NPs with different physicochemical properties or different experimental conditions are likely to produce different results. Interaction between particles and biomatrices is another important factor for toxicological consideration. Particles administered orally encounter diverse biological matrices, such as gastrointestinal (GI) fluids and blood, and these interactions lead to the formation of particle–biomatrix corona, which can alter their physicochemical property, biological interaction, and biological fate [13,14]. In particular, NPs–protein corona formation has been well reported to affect cellular responses. For example, it was reported that the ZnO NPs–serum protein interaction influences cytotoxicity, showing lower or higher cytotoxicity when NPs were dispersed in serum proteins [11,15,16]. The aggregation of di-block copolymer NPs was found to be induced by fibrinogen, while the adsorption of albumin and complement component 3 (C3) protein on the surface of NPs triggered the activation of the immune complement cascade [17]. In addition, it was reported that the NPs–plasma protein interaction can be implicated in immunological recognition, molecular targeting, biodistribution, and intracellular uptake [18]. The majority of studies on this topic have focused on the determination of NPs interactions with plasma proteins, though these interactions are surely affected by particle size. Indeed, ZnO NPs were reported to exhibit a higher cytotoxicity and inflammation response than micro ZnO in human monocytes, and their size-dependent cytotoxicity toward human lung epithelial cells was also demonstrated [19,20,21]. Moreover, the question as to whether NPs interactions with biomatrices lead to positive or negative effects on biological systems remains to be answered.

In the present study, we explored the interactions between ZnO particles and biological fluids (gastric fluids, intestinal fluids, and plasma) with respect to particle size (bulk vs. nano), and examined the physicochemical characteristics (hydrodynamic radius, zeta potential, and dissolution property) of bulk and nano ZnOs in vitro simulated biological fluids and ex vivo rat-extracted fluids. Particle–protein interactions were evaluated by measuring the protein fluorescence quenching ratio in the presence of particles, and proteomic analysis was further conducted to identify the plasma proteins adsorbed on the surface of bulk and nano ZnOs, respectively. Finally, we investigated the effect of particle size on the intestinal transport mechanism.

## 2. Results

### 2.1. Characterization of Bulk and Nano ZnOs

**Figure 1:**
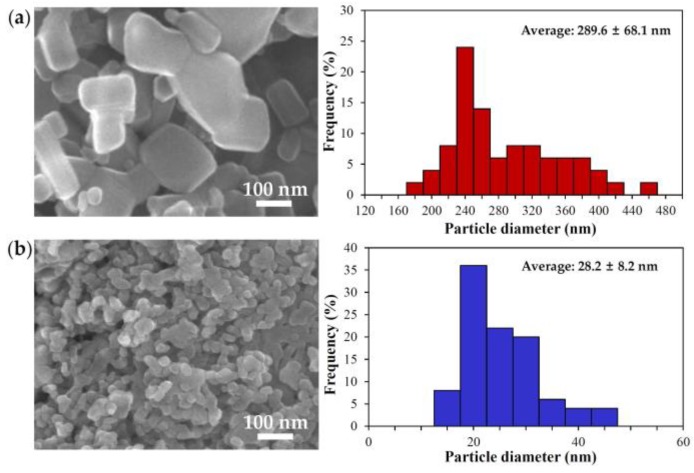
Scanning electron microscopic (SEM) images and size distribution of (**a**) bulk ZnO and (**b**) nano ZnO. Particle size distribution was determined by randomly selecting 200 particles from the SEM images.

The particle size, morphology, and size distribution of ZnOs were determined by scanning electron microscopy (SEM). The images showed that nano ZnO particles were spherical and had a narrow size distribution, whereas bulk ZnO particles were more irregular with a rectangle- or square-like shape and wider size distribution (Figure 1). The average primary particle sizes of bulk and nano ZnOs, as determined from the SEM images, were 289.6 ± 68.1 and 28.2 ± 8.2 nm, respectively. However, dynamic light scattering (DLS) data revealed that both particles agglomerated or aggregated when suspended in distilled water (DW), showing 3453.3 ± 278.0 and 1976.0 ± 198.7 nm for bulk and nano ZnOs, respectively (Table 1, Figure S1). On the other hand, the zeta potential values of the bulk and nano ZnOs were similar, showing 17.5 ± 1.6 and 16.0 ± 1.0 mV for the former and the latter, respectively, without statistical difference (*p* > 0.05, Table 2).

### 2.2. Changes in the Physicochemical Properties of ZnOs in Simulated Biofluids

The zeta potentials and hydrodynamic radii of ZnO particles in simulated biological fluids were measured in order to investigate changes in their physicochemical properties after interactions. Table 1 shows that the hydrodynamic radii of bulk ZnO gradually decreased in simulated intestinal and plasma fluids, while a significant decrease was only found in simulated gastric fluid during 1 h. In particular, a remarkable decrease was observed under the simulated plasma condition upon incubation. On the other hand, the overall increase in the hydrodynamic radii of nano ZnO was found under simulated gastric and intestinal conditions, whereas the hydrodynamic size decreased with incubation in plasma fluid versus that in DW.

The negative zeta potential values for both bulk and nano ZnOs were measured in all biological fluids (Table 2), though negative surface charges were significantly greater for bulk ZnO than for nano ZnO under all conditions tested.

### 2.3. Dissolution Properties of ZnOs in Simulated Biofluids

**Figure 2:**
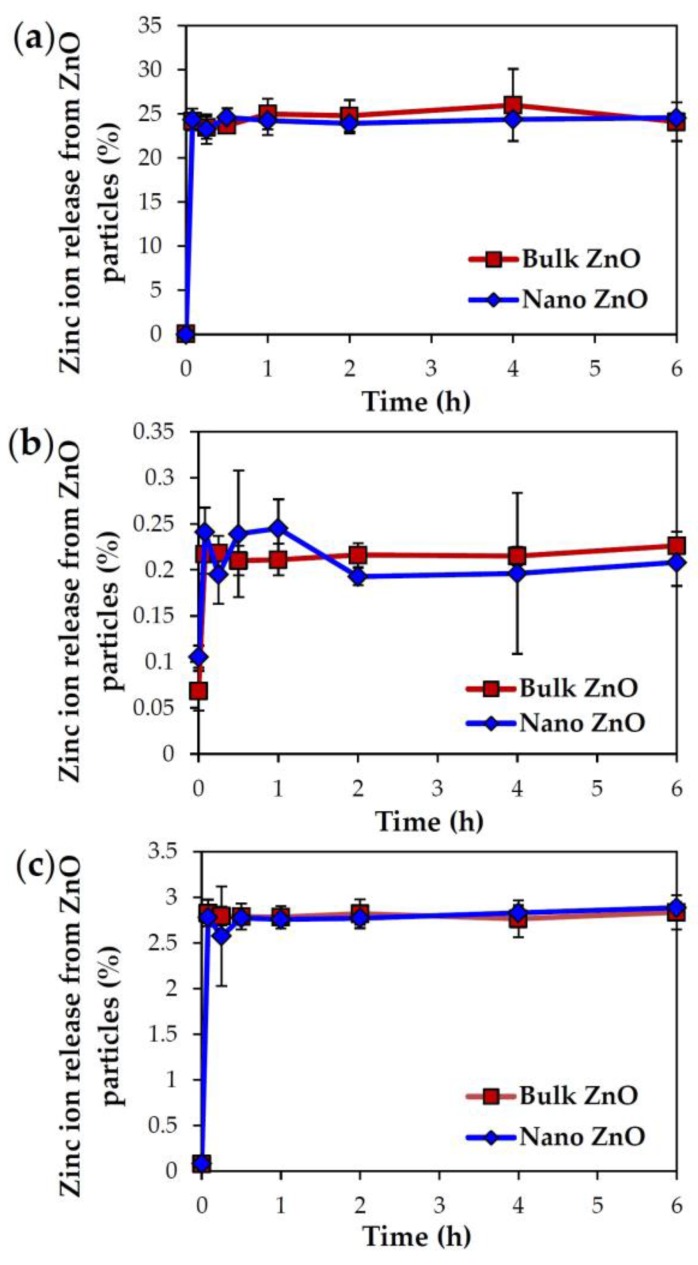
In vitro dissolution properties of ZnOs in simulated (**a**) gastric, (**b**) intestinal, and (**c**) plasma fluids. No significant differences between particle sizes were found (*p* > 0.05).

The solubility of ZnO particles was evaluated in simulated gastric, intestinal, and plasma fluids in order to elucidate their biological fate when administered orally. It was found that 24.5% and 24.2% of bulk and nano ZnOs, respectively, dissolved into zinc ions in simulated gastric fluid (Figure 2a). Meanwhile, the respective solubilities of ZnOs were ~0.2% and 2.8% under simulated intestinal and plasma conditions (Figure 2b,c), respectively. In all cases, no significant differences between particle sizes were found (*p* > 0.05).

**Figure 3:**
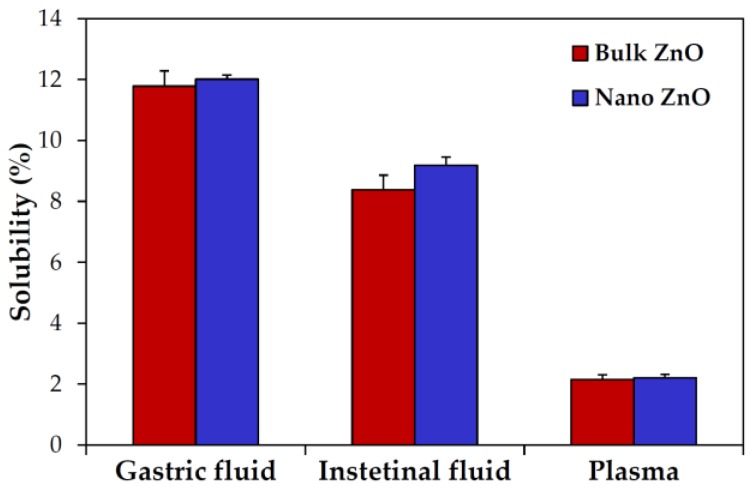
Ex vivo dissolution properties of ZnOs in rat-extracted gastrointestinal fluids and plasma after incubation for 30 min. No significant differences between particle sizes were found (*p* > 0.05).

On the other hand, the ex vivo solubility of ZnO particles was also investigated in rat-extracted biological fluids; ~12%, ~9%, and 2% solubilities were found in gastric fluid, intestinal fluid, and plasma, respectively (Figure 3). Particle size was not found to influence ex vivo solubility (*p* > 0.05).

### 2.4. ZnO Interactions with Proteins in Simulated Biofluids

**Figure 4:**
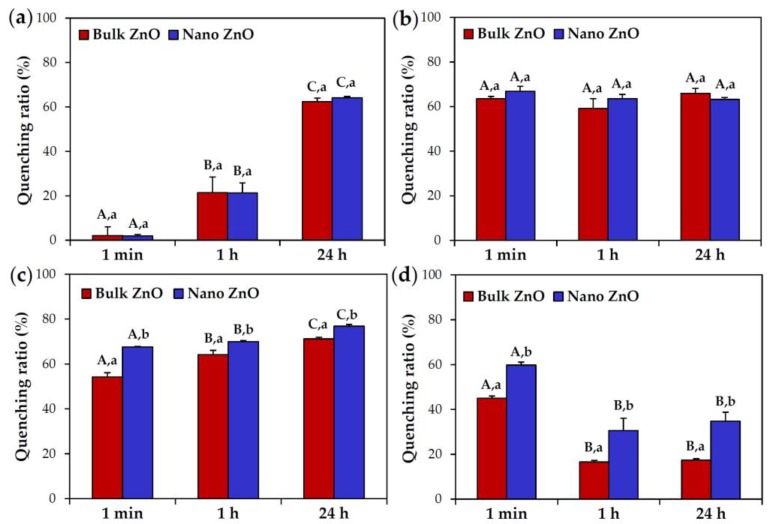
Protein fluorescence quenching ratios of bulk and nano ZnOs in simulated (**a**) gastric and (**b**) intestinal fluids and in (**c**) plasma containing bovine serum albumin (BSA) or (**d**) fibrinogen. Different letters capital (A,B,C) and lower case (a,b) letters indicate significant differences between incubation times and between bulk and nano ZnOs, respectively (*p* < 0.05).

When particle interactions with proteins were evaluated in simulated biofluids by measuring the protein fluorescence quenching ratio, a gradual and dramatic increase in fluorescence quenching by bulk and nano ZnOs was found in the simulated gastric fluid upon incubation time, but no significant difference was observed between particle sizes (Figure 4a) (*p* > 0.05). The fluorescence ratios of both bulk and nano ZnO reached ~64% after incubation for 24 h. Relatively high fluorescence quenching (~66%) was induced just after adding both particle types to simulated intestinal fluid and this was maintained for 24 h (Figure 4b). On the other hand, particle interactions with plasma were simulated using phosphate buffered saline (PBS) containing bovine serum albumin (BSA) or fibrinogen, the most abundant plasma proteins. A gradual increase in the fluorescence quenching ratio was observed under simulated plasma condition containing BSA (Figure 4c).

In particular, a statistically high fluorescence quenching ratio was found in the presence of nano ZnO versus bulk ZnO. Nano ZnO also interacted more strongly with simulated plasma fluid containing fibrinogen than bulk ZnO, but the highest fluorescence quenching was observed immediately after adding particles and subsequently decreased (Figure 4d). Meanwhile, slight red shifts by both bulk and nano ZnOs were observed in simulated gastric and intestinal fluids after incubation for 24 h (Figure S2).

### 2.5. ZnO Plasma–Protein Corona

**Figure 5:**
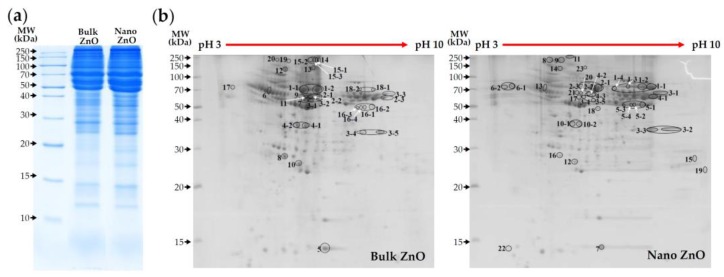
Plasma protein-binding profiles of bulk and nano ZnOs separated by (**a**) one-dimensional and (**b**) two-dimensional gel electrophoresis.

Rat plasma proteins bound to ZnO particles were quantitatively analyzed by Bradford assay before gel electrophoresis. Higher amount of proteins was found to be adsorbed on nano ZnO than bulk ZnO, demonstrating a total of 1544 and 2152 μg adsorbed proteins for bulk and nano ZnOs, respectively. Plasma proteins adsorbed on the surface of ZnO particles were determined by one-dimensional (1D) gel electrophoresis (Figure 5a). The results show that the patterns of proteins bound to bulk and nano ZnOs differ. In particular, a much larger amount of proteins was adsorbed on nano ZnO. Further protein analysis by two-dimensional (2D) gel electrophoresis showed different binding profiles between two particles (Figure 5b). As expected, a more intense interaction between nano ZnO and plasma proteins were found.

The protein corona that formed around the particles were identified by mass spectrophotometry (MS) according to protein molecular weight (MW) and isoelectric point (pI). The most abundant plasma proteins identified in the particle–protein coronas are listed in Table 3. A total of 20 and 23 proteins were determined to be adsorbed on bulk and nano ZnOs, respectively. Among them, 19 proteins were commonly found in coronas, regardless of particle size. Serum albumin and fibrinogen were the most abundant two proteins in the corona formed on both bulk and nano ZnOs. Fibronectin was also commonly and frequently found in particle–protein corona, regardless of particle size. However, the plasma protein-binding profiles between bulk and nano ZnOs differed. In particular, fibronectin 1 isoform CRA-b was only detected in the bulk ZnO-protein corona, while complement C1q subcomponent subunit B precursor, complement C3 precursor, SWItch/sucrose non-fermentable (SWI/SNF)-related matrix-associated actin-dependent regulator of chromatin subfamily D member 3, and keratin K6 were only identified in nano ZnO-protein corona.

### 2.6. Intestinal Transport Mechanism

**Figure 6:**
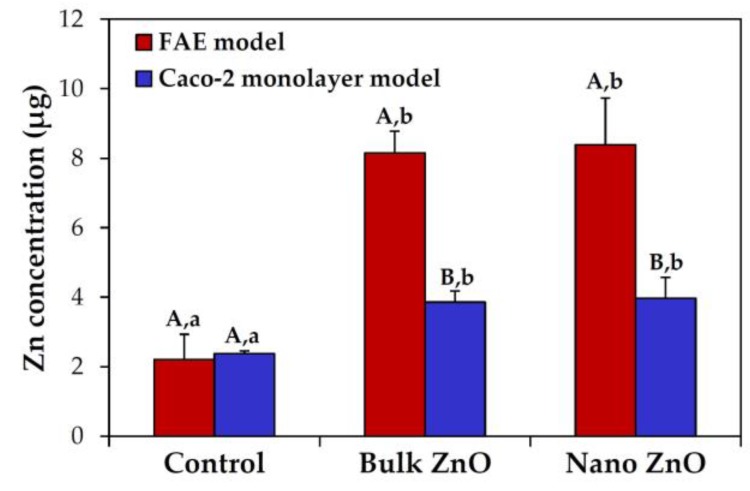
Intestinal transport of bulk and nano ZnOs using an in vitro human follicle-associated epithelium (FAE) model and a Caco-2 monolayer model. Different capital (A,B) and lower case (a,b) letters indicate significant differences between the FAE and Caco-2 monolayer models and among untreated control, bulk ZnO, and nano ZnO, respectively (*p* < 0.05).

The intestinal transport mechanism of bulk and nano ZnOs was determined using an in vitro human follicle-associated epithelium (FAE) model and Caco-2 monolayers, which represent microfold (M) cells in Peyer’s patches and intestinal tight junction barriers, respectively. The result demonstrated that both different-sized ZnOs were primarily transported by M cells, and a slight increase in both particle transports through Caco-2 monolayers was also found (Figure 6). However, transported amounts were not found to be dependent on particle size (*p* > 0.05).

## 3. Discussion

In the present study, we evaluated ZnO interactions with in vitro and ex vivo biological matrices, such as GI fluids and plasma, with respect to particle size (bulk vs. nano) in order to elucidate the effects of particle size on biological interactions. The particle size (289.6 ± 68.1 nm) and hydrodynamic size (3453.3 ± 278.0 nm) of bulk ZnO were quite different from those of nano ZnO (28.2 ± 8.2 and 1976.0 ± 198.7 nm), and both particles formed agglomerates or aggregates in aqueous solution (Table 1, Figure S1).

The hydrodynamic radii and zeta potential values of the bulk and nano ZnOs changed in all biological fluids, indicating particle interaction with biomatrices (Table 1 and Table 2). In particular, a significant decrease in the hydrodynamic radii of both particles under simulated plasma conditions observed after incubation for 24 h suggests that plasma proteins facilitate particle dispersion (Table 1) [22,23,24]. It is worth noting that bulk and nano ZnOs formed agglomerates or aggregates in DW and biofluids. ZnO NPs were reported to agglomerate or aggregate in aqueous solution, but their hydrodynamic size remarkably decreased in the presence of BSA or serum protein [15,25,26,27]. Hence, it seems that ZnO particles form agglomerates, not aggregates, and their dispersion can be enhanced by particle–protein interaction. Meanwhile, the positive zeta potential values of bulk and nano ZnOs in DW became negative in all simulated biofluids (Table 2), indicating the interaction effect on surface characteristics of particles. In addition, significantly more negative zeta potentials were found for bulk ZnO than for nano ZnO in all biofluids, implying different interaction between particle sizes.

However, particle size did not affect solubility, as demonstrated by dissolutions of ~24%, 0.2%, and 2.8% in simulated gastric fluid, intestinal fluid, and plasma, respectively, for both particle sizes (Figure 2). The high dissolution properties of ZnO NPs under acidic and gastric conditions [28] and low solubility in neutral fluids have been well reported [29,30], which is in good agreement with our results. Further investigation on ex vivo solubility showed ~12%, ~9%, and 2% dissolution of both bulk and nano ZnOs in rat-extracted gastric, intestinal, and plasma fluids, respectively, without statistical significances between particle sizes (Figure 3). The in vitro and ex vivo solubility of both ZnOs differed, except in plasma. This lower ex vivo solubility of ZnOs in rat-extracted gastric fluid than in vitro may be due to the comparatively high pH of rat gastric fluid (pH~3.2 in rats and pH~1.5 in man) [31]. On the other hand, higher solubility was found in rat intestinal fluid than in simulated fluid, which suggests that the total amount of acidic gastric fluid in rat intestinal fluid was greater than in simulated intestinal fluid. Taken together, these results suggest that ZnO particles are primarily present in particulate forms after oral administration, regardless of particle size, although some portion can be dissolved into zinc ions. Interestingly, it would appear that particle size does not critically affect the solubility and biological fate of ZnOs, which is in line with the previously published finding that ZnO particle size (bulk vs. nano) had no effect on absorption following oral administration to rats [32].

Since all biofluids contain proteins, ZnO interaction with proteins was evaluated by measuring the protein fluorescence quenching ratio in the presence of particles. Fluorescence quenching was observed in all biological fluids (Figure 4), suggesting clear particle–protein interactions, although the quenching ratios depended on biofluid type. Similar particle interactions with GI fluids, regardless of particle size, imply a low effect of particle–protein interaction on oral absorption. On the other hand, the fact that red shifts were observed for both different-sized ZnOs in GI fluids (Figure S1) suggests conformational or structural changes of digestion enzymes, which could potentially affect the digestion and utilization of nutrients. It is worth noting that the quenching ratios of nano ZnO were significantly higher than those of bulk ZnO under plasma conditions containing BSA or fibrinogen, suggesting particle-size-dependent interactions in plasma. It seems that particle size can more critically affect particle–protein corona formation in plasma, than in GI fluid. Moreover, both ZnO size types interacted more strongly with BSA than with fibrinogen, which is in line with our proteomic results (Table 3).

When the particle–plasma protein corona was further investigated using a proteomic approach, an obvious particle-size-dependent difference was observed by 1D and 2D gel electrophoresis (Figure 5). Furthermore, a much larger amount and more protein types were found to be adsorbed on nano ZnO (23 kinds) than on bulk ZnO (20 kinds). MS results in the identification of particle–protein corona revealed that serum albumin was the most important protein forming the corona, followed by fibrinogen, regardless of particle size (Table 3). Fibronectin was also frequently found in the coronas of both bulk and nano ZnOs. Indeed, albumin, fibrinogen, and fibronectin have been reported to be abundantly adsorbed on NPs [33,34,35]. Albumin is the most abundant plasma protein and is responsible for colloid osmotic pressure, transportation, and detoxification [36,37]. Hence, the formation of ZnO–albumin corona could affect particle toxicity, distribution, and circulation time. Fibrinogen and fibronectin are both glycoproteins; fibrinogen is involved in the blood coagulation system [38,39] and fibronectin plays a role in cell adhesion, growth, and wound healing [35,40]. Thus, particle interactions with fibrinogen or fibronectin could affect innate immune response. The complementary system is an essential part of the immune system [41,42], which was only found in nano ZnO–plasma protein corona. These findings show that ZnO–plasma protein interactions are dependent on particle size and suggest that nano ZnO is more likely to affect immune response than bulk ZnO.

On the other hand, the intestinal transport mechanism was not influenced by particle size (Figure 6), indicating that bulk and nano ZnOs were transported through M cells and Caco-2 monolayers. ZnO particles were primarily transported by M cells, regardless of particle size. M cells found in Peyer’s patches in the small intestine are implicated in the transport of various molecules, including macromolecules and particles [43,44]. Indeed, the intestinal transportation of diverse NPs by M cells has been recently reported [45,46,47], which concurs with our results. It should be noted that ZnO particles can be also transported through intestinal tight junction barriers, as demonstrated by our Caco-2 monolayer model. This may be associated with their partial ionized fate, because non-ionized NPs under physiological conditions tend not to be transported through tight junctions [48,49]. Moreover, no significant differences in the intestinal transportation amount between particle sizes were found, which could explain the similar oral absorption between bulk ZnO and nano ZnO [32]. It appears that particle–protein interactions did not remarkably affect the intestinal transport mechanism and oral bioavailability, regardless of particle size, but particle size plays a role in interactions with plasma proteins in blood. Further study is required to elucidate the impact of ZnO NP–protein interactions on potential long-term toxicity.

## 4. Materials and Methods 

### 4.1. Materials

Nano ZnO (20 nm) and bulk ZnO (5 μm) were purchased from Sumitomo (Tokyo, Japan) and Sigma-Aldrich (St Louis, MO, USA), respectively. Each particle was dispersed in DW (5 mg/mL) for 30 min, just prior to experiments.

### 4.2. Characterization

Primary particle size and morphology were examined using SEM (FEIQUANTA 250 FEG, Hillsboro, OR, USA). The hydrodynamic radius and zeta potential were determined with a Zetasizer Nano Series (Malvern, Westborough, MA, USA). The measurements were performed at 25 °C by dispersing particles in DW or biofluids.

### 4.3. Preparation of Simulated Biofuids

Simulated gastric, intestinal, and plasma fluids were prepared for in vitro studies as previously described [50]. The simulated gastric fluid was prepared by dissolving 2 g sodium chloride (Samchun Chemical Co., Ltd., Pyeongtaek, Korea) and 3.2 g pepsin (Sigma-Aldrich, St Louis, MO, USA) in DW, and the pH was adjusted to 1.5 with 1 N hydrochloric acid (Duksan Pure Chemicals Co., Ltd., Ansan, Gyeonggi-do, Korea), and then made up to 1000 mL. For the simulated intestinal fluid, bile salt (87.5 mg) (Sigma-Aldrich, St Louis, MO, USA) and pancreatin (25 mg) (Sigma-Aldrich, St Louis, MO, USA) were added to the simulated gastric fluid, and then the pH was adjusted to 6.8 with saturated sodium bicarbonate solution (Sigma-Aldrich, St Louis, MO, USA). The simulated plasma fluid was prepared by dissolving 50 g of BSA (Sigma-Aldrich, St Louis, MO, USA) in PBS solution (NaCl 137 mM, KCl 2.7 mM, Na_2_HPO_4_ 10 mM, KH_2_PO_4_ 1.8 mM; Dongin Biotech. Co., Ltd., Seoul, Korea), and then made up to 1000 mL.

### 4.4. Animals and Preparation of Rat-Extracted Biofluids

Five-week-old female Sprague Dawley (SD) rats weighing around 150 g were obtained from Nara Biotech Co., Ltd. (Seoul, Korea) and acclimated to environments for seven days before experiment. Animals were housed in plastic animal cages in a ventilated room maintained at 20 ± 2 °C and 60 ± 10% relative humidity under a 12 h light/dark cycle. Water and commercial laboratory complete feed for rats were provided *ad libitum*. All animal experiments were performed in accordance with the approved animal protocol and guideline established by the Animal and Ethics Review Committee of Seoul Women’s University (IACUC-2016A-3).

Rat biofluids, such as GI fluids and plasma, were obtained as previously reported [50]. Briefly, stomachs and small intestines were collected and rinsed with saline, and then gastric and intestinal fluids were obtained by centrifugation at 16,000× *g* for 15 min at 4 °C. To obtain plasma, blood sample was collected via tail vein using a catheter, and centrifuged at 16,000× *g* for 3 min at 4 °C.

### 4.5. In Vitro and Ex Vivo Dissolution Properties of ZnO in Biofluids

Each particle (bulk and nano ZnOs) suspension was added to simulated and rat-extracted biofluids (5 mg/mL) and incubated with gentle shaking (180 rpm) at 37 °C. After designated incubation times, supernatants were collected by centrifugation (16,000× *g*) for 15 min. Pre-digestion of the collected supernatants was performed with 10 mL of 60% ultrapure nitric acid and 0.5 mL of H_2_O_2_ at 180 °C until samples were completely digested. Solutions were diluted with 2.5 mL of distilled and deionized water (DDW) and quantitative analysis of the dissolved Zn from ZnO was carried out using inductively coupled plasma–atomic emission spectroscopy (ICP-AES, JY2000 Ultrace, HORIBA Jobin Yvon, Longjumeau, France).

### 4.6. Fluorescence Quenching Measurement

To determine particle–protein interaction, a fluorescence quenching measurement was performed in two different simulated plasma fluids. BSA (Sigma-Aldrich, St Louis, MO, USA) or fibrinogen (Sigma-Aldrich, St Louis, MO, USA) was added to PBS at concentration of 1 mg/mL. ZnO particle suspensions (100 μL of 5 mg/mL) were added in 1 mL of simulated solutions and incubated for 1 min, 1 h, and 24 h at 37 °C with gentle shaking (180 rpm). A protein fluorescence quenching assay was carried out using a spectrophotometer (Molecular Devices, LLC., Sunnyvale, CA, USA). Fluorescence spectra were measured at 300–420 nm using an excitation wavelength of 280 nm. Protein fluorescence quenching ratios were calculated as (I0-I)/I0, where I0 and I stand for fluorescence intensities in the absence and presence of ZnO particle suspensions, respectively.

### 4.7. 1D and 2D Gel Electrophoresis

Particle suspensions of bulk and nano ZnOs (100 μL of 50 mg/mL) were incubated with 1 mL of rat plasma at 37 °C with gentle shaking (180 rpm) for 1 h, and then centrifuged at 23,000× *g* for 1 h at 4 °C in order to separate unbound proteins. The precipitates were washed three times with DDW. The 1D gel electrophoresis was carried out by suspending samples in rehydration buffer (7 M urea, 2 M thio-urea, 4% 3-[(3-cholamido-propyl)dimethylammonio]-1-propanesulfonate (CHAPS), 2.5% dithiothreitol) containing protease inhibitor cocktail (Roche Molecular Biochemicals, Indianapolis, IN, USA), and by vortexting overnight. After centrifugation at 15,000× *g* for 20 min, the lysates were re-suspended in sodium dodecyl sulfate (SDS) sample buffer (2% SDS, 0.1% bromophenol blue, 10% glycerol, 0.5% β-mercaptoethanol in 50 mM Tris-HCl, pH 6.8), and then heated at 95 °C for 5 min. After cooling to room temperature, samples (30 μg of protein) were loaded into 15% SDS-polyacrylamide (PAGE) gels. Protein concentrations were determined by Bradford method (Bio-Rad, Hercules, CA, USA).

For 2D gel electrophoresis, pH 3–10 immobilized pH gradient (IPG) strips (GE Healthcare Life Sciences, Pittsburgh, PA, USA) were rehydrated in swelling buffer (7 M urea, 2 M thiourea, 0.4% (*w*/*v*) dithiothreitol, and 4% (*w*/*v*) CHAPS). The protein lysates (700 μg) were loaded into the rehydrated IPG strips using an IPGphor III (GE Healthcare Life Sciences). 2D separation was performed on 14% (*v*/*v*) SDS-PAGE gels. After gel fixation for 1 h in 40% (*v*/*v*) methanol containing 5% (*v*/*v*) phosphoric acid, the gels were stained with Coomassie blue G-250 solution (ProteomeTech, Seoul, Korea), and destained in 1% (*v*/*v*) acetic acid. Images were acquired with an Image Scanner III (Bio-Rad, Hercules, CA, USA).

### 4.8. Identification of Proteins by Liquid Chromatography-Mass Spectrometry/Mass Spectrometry

Image analysis was carried out using an Image Master^TM^ 2D Platinum software (GE Healthcare Life Science, Pittsburgh, PA, USA). To compare the densities of protein spots induced by bulk or nano ZnOs, more than 25 spots were landmarked and normalized. In-gel digestion of protein spots from Coomassie Blue stained gels was performed as previously described [51]. Prior to mass spectrometric analysis, the peptide solutions were desalted using a reversed-phase column [52]. The eluted peptides were analyzed by liquid chromatography-mass spectrometry/mass spectrometry (LC-MS/MS) on a nano ACQUITY UPLC (Waters, Milford, MA, USA) directly coupled to a Finnigan LCQ DECA iontrap mass spectrometer (Thermo Scientific, Waltham, MA, USA). Spectra were acquired and processed using the MASCOT software (Matrix Science, London, UK). The individual spectra from MS/MS were processed using a SEQUEST software (Thermo Quest, San Jose, CA, USA). Only significant hits as defined by the MASCOT software (Matrix Science, London, UK) probability analysis were taken.

### 4.9. Three Dimensional (3D) Cell Culture for FAE Model

ZnO particle transport by M cells was investigated using an in vitro human intestinal FAE model, as previously described [53]. Human intestinal epithelial Caco-2 cells and non-adherent human Burkitt’s lymphoma Raji B cells (Korean Cell Line Bank, Seoul, Korea) were grown in minimum essential medium (MEM; Welgene Inc., Gyeongsangbuk-do, Korea) and Roswell Park Memorial Institute (RPMI) 1640 medium (Welgene Inc.), respectively, supplemented with 10% fetal bovine serum, 1% non-essential amino acids, 1% L-glutamine, 100 units/mL penicillin, and 100 μg/mL streptomycin at 37 °C under 5% CO_2_ atmosphere. After coating Transwell^®^ polycarbonate inserts (SPL Lifescience, Gyeonggi-do, Korea) with Matrigel™ matrix (Becton Dickinson, Bedford, MA, USA) for 2 h, supernatants were removed, and then inserts were washed with DMEM. Caco-2 cells (1 × 10^6^ cells/well) were seeded in the apical side and grown for 14 days. Lymphoma Raji B cells (1 × 10^6^ cells/well) were added to the basolateral side, and maintained for five days. Apical medium was then replaced with ZnO suspensions (16.25 μg/mL), and incubation continued for 6 h. The transported amounts of ZnO particles were determined by measuring total Zn levels in the basolateral side using ICP-AES (JY2000 Ultrace, HORIBA Jobin Yvon). Pre-digestion for the ICE-AES analysis was performed in the same manner as described in “In vitro and ex vivo dissolution properties”.

### 4.10. 3D Cell Culture for Intestinal Epithelial Monolayers

The transport of ZnO particles by the intestinal epithelium monolayer was evaluated using an in vitro Caco-2 monoculture system. Caco-2 cells (4.5 × 10^5^ cells/well) were seeded on the upper insert side in the same manner as described in the FAE model, and then cultured for 21 days. After replacing the apical medium of cell monolayers with ZnO suspensions (16.25 μg/mL), incubation continued for 6 h. The transported concentrations of ZnO particles were determined in the same manner as described in the FAE model.

### 4.11. Statistical Analysis

Results were presented as means ± standard deviation. Experimental values were compared with each other. One-way analysis of variance (ANOVA) with Tukey’s Test in SAS Ver.9.4 (SAS Institute Inc., Cary, NC, USA) was used to determine the significances between experimental groups. Statistical significance was accepted for *p* values < 0.05.

## 5. Conclusions

In this study, ZnO interactions with biological fluids were clearly demonstrated in terms of changes in the physicochemical properties, solubility, fluorescence quenching, and particle–plasma protein corona formation. Bulk and nano ZnO were found to interact differently with biomatrices, in particular, nano ZnO exhibited lower negative surface charges and had higher fluorescence quenching ratios under simulated plasma condition. More abundant plasma proteins were determined to be adsorbed on nano ZnO than on bulk ZnO. In particular, complement C was only identified in nano ZnO-plasma protein corona, while serum albumin, fibrinogen, and fibronectin seemed to play roles in corona formation, regardless of particle size. However, particle solubility and the intestinal transport mechanism did not appear to be influenced by particle size. Further study is required to elucidate the effect of NP interaction with biomatrices on potential toxicity and nutrient absorption after long-term exposure.

## Figures and Tables

**Table 1:** Hydrodynamic radii of bulk and nano ZnOs in different simulated biological fluids.

**Hydrodynamic radius (nm)**	**Size**	**Time**	**DW**	**Gastric Fluid**	**Intestinal Fluid**	**Plasma**
	Bulk ZnO	1 min	3453.3 ± 278.0 ^A,a^	2161.0 ± 257.0 ^B,b^	3224.5 ± 180.0 ^A,a^	3327.3 ± 268.8 ^A,a^
		1 h		2463.0 ± 235.3 ^B,b,c^	2828.5 ± 158.7 ^A,b^	2184.3 ± 203.8 ^B,c^
		6 h		3176.0 ± 272.4 ^A,a^	2585.0 ± 84.9 ^B,b^	1735.8 ± 114.0 ^C,c^
		24 h		3237.3 ± 81.8 ^A,a^	2227.5 ± 139.0 ^C,b^	1473.8 ± 51.4 ^C,c^
	Nano ZnO	1 min	1976.0 ± 198.7 ^A,b^	2060.8 ± 41.4 ^A,B,a^	3009.8 ± 200.4 ^B,c^	2485.3 ± 226.9 ^B,b,^*
		1 h		2365.0 ± 188.9 ^B,a,b^	3079.8 ± 206.5 ^B,c^	2439.0 ± 144.8 ^B,b^
		6 h		2993.8 ± 203.2 ^C,c^	2435.0 ± 162.3 ^C,b^	1761.8 ± 250.0 ^A,C,a^
		24 h		3180.8 ± 81.3 ^C,c^	2300.5 ± 131.7 ^A,C,b^	1502.3 ± 187.8 ^C,a^

Different capital (A,B,C) and lower case (a,b,c) letters indicate significant differences between incubation times and between simulated biofluid types, respectively (*p* < 0.05). * indicates significant differences compared to bulk ZnO (*p* < 0.05).

**Table 2:** Zeta potentials of bulk and nano ZnOs in different simulated biological fluids.

**Zeta potential (mV)**	**Size**	**Time**	**DW**	**Gastric Fluid**	**Intestinal Fluid**	**Plasma**
	Bulk ZnO	1 min	17.5 ± 1.6 ^A,a^	−17.4 ± 0.5 ^B,b^	−23.4 ± 0.6 ^B,c^	−27.5 ± 0.9 ^B,d^
		1 h		−16.9 ± 0.6 ^B,b^	−25.9 ± 1.8 ^C,c^	−27.9 ± 0.9 ^B,c^
		6 h		−16.9 ± 0.9 ^B,b^	−27.1 ± 0.9 ^C,D,c^	−28.5 ± 0.4 ^B,c^
		24 h		−16.6 ± 0.4 ^B,b^	−28.6 ± 0.7 ^D,c^	−27.9 ± 0.7 ^B,c^
	Nano ZnO	1 min	16.0 ± 1.0 ^A,a^	−14.8 ± 0.7 ^B,b,^*	−19.0 ± 0.6 ^B,c,^*	−23.1 ± 1.0 ^B,d,^*
		1 h		−13.5 ± 0.8 ^B,b,^*	−20.6 ± 0.9 ^C,c,^*	−22.8 ± 0.9 ^B,d,^*
		6 h		−13.8 ± 0.7 ^B,b,^*	−20.7 ± 0.6 ^C,c,^*	−24.6 ± 0.8 ^B,C,d,^*
		24 h		−13.6 ± 1.0 ^B,b,^*	−17.2 ± 2.1 ^B,c,^*	−26.2 ± 0.7 ^C,d,^*

Different capital (A,B,C,D) and lower case (a,b,c,d) letters indicate significant differences between incubation times and between simulated biofluid types, respectively (*p* < 0.05). * indicates significant differences compared to bulk ZnO (*p* < 0.05).

**Table 3:** List of the most abundant plasma proteins adsorbed on bulk and nano ZnOs as determined by liquid chromatography-mass spectrometry/mass spectrometry (LC-MS/MS).

MW (kDa)	pI	Bulk ZnO	No.	Nano ZnO	pI	MW (kDa)
68.7	6.09	Serum albumin	1	Serum albumin precursor	6.09	68.8
54.3	7.89	Fibrinogen B beta chain	2	Serum albumin	6.09	68.7
60.5	7.56	Fibrinogen alpha subunit	3	Fibrinogen alpha subunit	7.56	60.5
167.2	6.46	Alpha-1-macroglobulin	4	Fibrinogen B beta chain	7.89	54.3
15.7	5.77	Prealbumin	5	Fibrinogen gamma chain precursor	5.85	49.7
45.7	5.37	Serine protease inhibitor A3K	6	Vitronectin	5.68	54.7
60.7	6.56	Fibrinogen alpha chain precursor	7	Prealbumin	5.77	15.7
26.2	5.50	Serum amyloid P-component precursor	8	Alpha-1-inhibitor 3	5.70	163.8
87.0	5.57	Fibrinogen alpha chain isoform X2	9	Fibronectin isoform X3	5.61	262.8
103.6	6.08	Inter-alpha-inhibitor H4 heavy chain	10	Alpha-1-macroglobulin	6.46	167.2
54.2	7.90	Fibrinogen beta chain precursor	11	Fibronectin isoform X2	5.54	262.8
254.4	5.27	Fibronectin isoform X6	12	Inter-alpha-inhibitor H4 heavy chain	6.08	103.6
272.6	5.50	Fibronectin	13	Serine protease inhibitor A3K	5.37	45.7
262.8	5.54	Fibronectin isoform X2	14	Fibronectin isoform X6	5.27	254.4
253.2	5.47	Fibronectin 1 isoform CRA-b	15	Complement C1q subcomponent subunit B precursor	9.13	26.6
49.7	5.85	Fibrinogen gamma chain precursor	16	Serum amyloid P-component precursor	5.50	26.2
54.7	5.68	Vitronectin	17	Fibrinogen alpha chain precursor	6.56	60.7
68.8	6.09	Serum albumin precursor	18	Complement C3 precursor	6.06	186.4
262.8	5.61	Fibronectin isoform X3	19	SWI/SNF-related matrix-associated actin-dependent regulator of chromatin subfamily D member 3	9.36	53.6
163.8	5.70	Alpha-1-inhibitor 3	20	Fibrinogen beta chain precursor	7.90	54.2
			21	Fibrinogen alpha chain isoform X2	5.57	87.0
			22	Keratin K6	3.10	5.6
			23	Fibronectin	5.50	272.6

Proteins marked in bold font were only found in bulk or nano ZnO corona. The list of proteins above is presented in intensity order as determined by 2D gel electrophoresis. Abbreviations: MW, molecular weight; kDa, kilo dalton; pI, isoelectric point; No, number.

## Supplementary Materials

The following are available online at www.mdpi.com/2079-4991/7/11/377/s1, Figure S1: Representative size distribution of (a,c,e) bulk ZnO and (b,d,f) nano ZnO, measured by dynamic light scattering (DLS), in simulated (a,b) gastric, (c,d) intestinal, and (e,f) plasma fluids; Figure S2: Fluorescence spectra of simulated (a,b) gastric fluid, (c,d) intestinal fluid, (e,f) plasma containing bovine serum albumin (BSA, plasma-BSA), and (g,h) plasma containing fibrinogen (plasma-fibrinogen) in the absence or presence of bulk ZnO or nano ZnOs.
