# Supplementary material for: ZnO Interactions with Biomatrices: Effect of Particle Size on ZnO-Protein Corona
Source: Nanomaterials (Basel). 2017 Nov 6;7(11):377. doi: 10.3390/nano7110377 (PMC5707594; doi:10.3390/nano7110377)
Supplement: Supplementary file 1 [file nanomaterials-07-00377-s001.pdf]

Supplementary materials

ZnO Interactions with Biomatrices: Effect of Particle Size on ZnO-Protein Corona

Jin Yu, Hyeon-Jin Kim, Mi-Ran Go, Song-Hwa Bae and Soo-Jin Choi *

Department of Applied Food System, Major of Food Science & Technology, Seoul Women's University, Seoul 01797, Korea; ky5031@swu.ac.kr (J.Y.); kimhj043@naver.com (H.-J.K.); miran8190@naver.com (M.-R.G.); songhwa29@naver.com (S.-H.B.)

* Correspondence: sjchoi@swu.ac.kr; Tel.: +82-2-970-5634; Fax: +82-2-970-5977

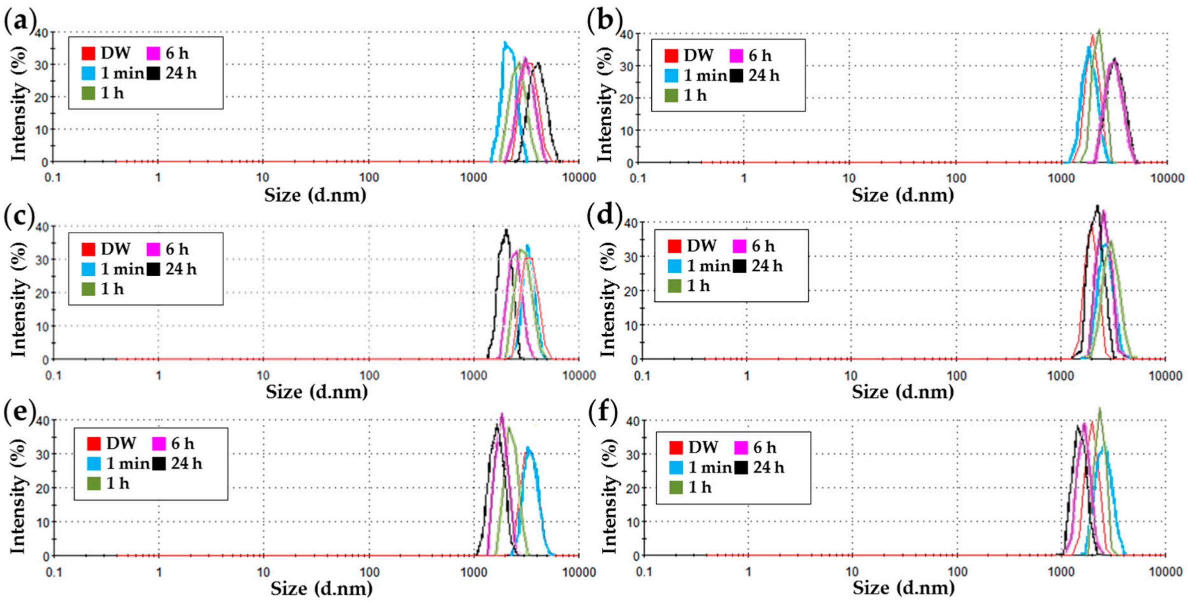

Figure S1. Hydrodynamic size distribution of (**a**,**c**,**e**) bulk ZnO and (**b**,**d**,**f**) nano ZnO in simulated (**a**–**b**) gastric fluid, (**c**–**d**) intestinal fluid, and (**e**–**f**) plasma measured by Zetasizer Nano Series.

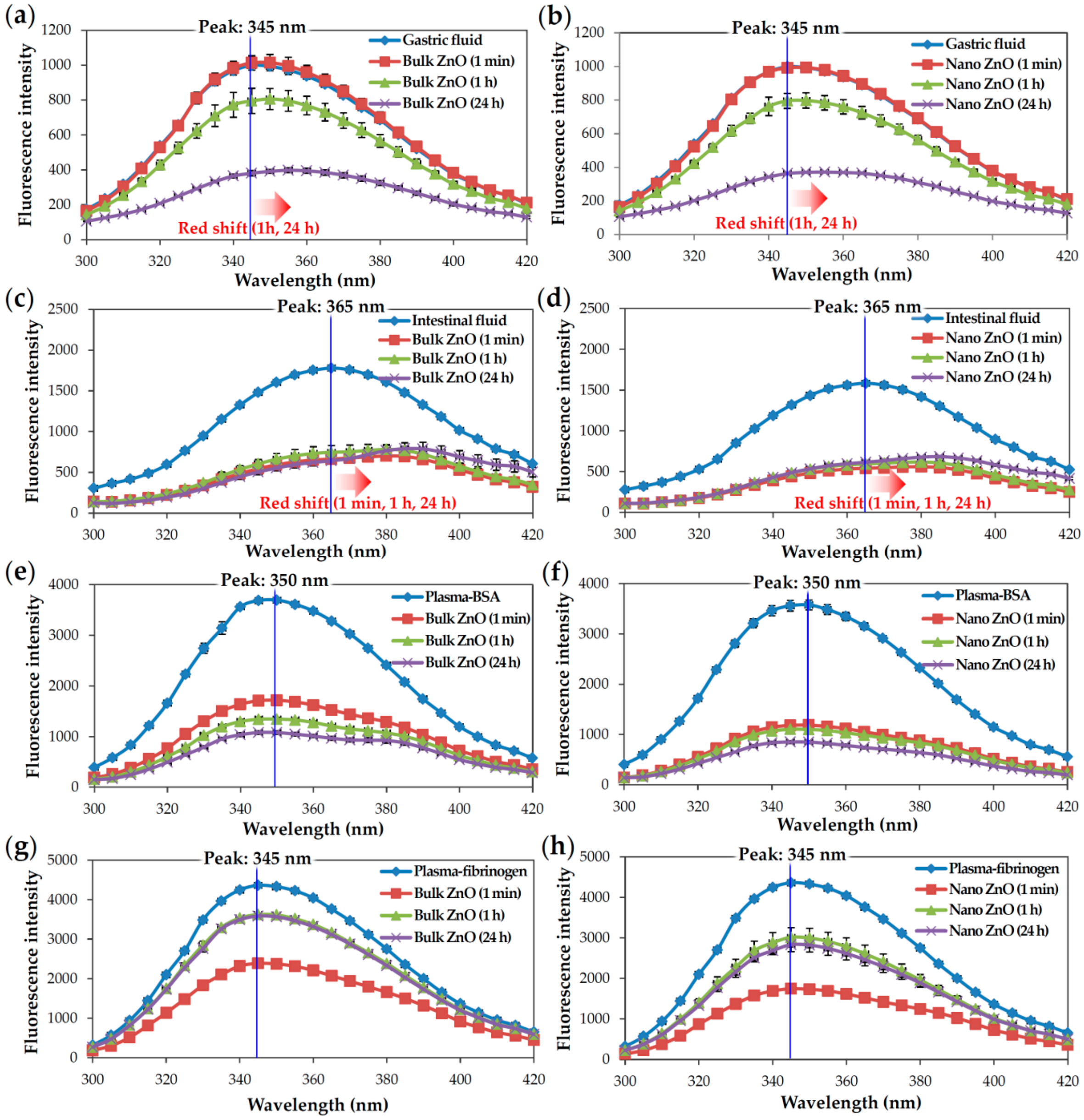

Figure S2. Fluorescence spectra of simulated (**a–b**) gastric fluid, (**c–d**) intestinal fluid, (**e–f**) plasma containing bovine serum albumin (BSA, plasma-BSA), and (**g–h**) plasma containing fibrinogen (plasma-fibrinogen) in the absence or presence of bulk ZnO or nano ZnOs.
